# Interfacial Structure and Bonding Properties of Ag/Cu Through-Layered Composite Fabricated by Dual-Face Hot-Roll Inlaying Process

**DOI:** 10.3390/ma18245580

**Published:** 2025-12-12

**Authors:** Yong Wang, Quanzhen Yang, Kunshan Guo, Tianhao Liu, Xue Zhao, Lei Huang, Haiguang Ruan, Xiaorong Zhou, Yi Chen

**Affiliations:** 1 College of Materials Science & Engineering, Chongqing University, Chongqing 400045, China; y717925821@163.com (Q.Y.); yichen@cqu.edu.cn (Y.C.); 2 National Engineering Research Center for Magnesium Alloys, Chongqing University, Chongqing 400045, China; 3 International Joint Laboratory for Light Alloys (MOE), Chongqing University, Chongqing 400045, China; 4 Chongqing Chuanyi Metallic Functional Materials Co., Ltd., Chongqing 400702, China; darcy8023@163.com (K.G.); 13368098397@163.com (T.L.); zzxxxx99602@163.com (X.Z.); 15823162713@163.com (L.H.); rsea828@163.com (H.R.); zxrong2004@163.com (X.Z.)

**Keywords:** Ag/Cu through-layered composite, dual-face hot-roll inlaying, interfacial structure, interdiffusion, bonding strength, resistance

## Abstract

**Highlights:**

**What are the main findings?**

- A novel dual-face hot-roll inlaying process was developed to fabricate a Ag/Cu through-layered composite. The Ag and Cu layers had the same textural components (copper, brass, and S-type components). However, no well-matched crystallographic orientation relationship was identified at the Ag/Cu interface.
- The width of the elemental interdiffusion layer is generally less than 2 μm. The Ag/Cu interface bonding strength surpasses the tensile strength of Ag (260 MPa), and each interface contributes an increase of 1.1% to the electrical resistivity of the composite.

**What is the implication of the main finding?**

- This Ag/Cu through-layered composite is a promising candidate for use as a substitute for pure Ag in the fabrication of melt elements in fuses, and it is commercially available.

**Abstract:**

A novel dual-face hot-roll inlaying technique was developed to fabricate a Ag/Cu through-layered composite for use in melt elements for fuse production, including two stages of grooving in a Cu strip followed by separate inlaying of Ag strips at the same positions on the opposite surfaces. The microstructure was characterized using field emission scanning electron microscopy (FE-SEM), electron probe microanalysis (EPMA), X-ray diffraction (XRD), and selective area electron diffraction (SAED). The Ag/Cu interfaces are flat and well bonded, with an elemental interdiffusion layer of less than 2 μm. The same textural components—copper, brass, and S-type components—were identified in both the Ag and Cu layers. However, no well-matched crystal orientation relationship between Ag and Cu was detected at the interface. Moreover, tensile properties and electrical resistance were measured to evaluate the bonding strength and conductivity of the interface. It was found that Ag/Cu bonding strength surpassed the tensile strength of Ag, i.e., 260 MPa. While the total elongation is less than 1%, the Ag layer exhibits excellent plasticity, with a section shrinkage over 90%. Compared with the calculated resistivity with a series circuit model, the tested value of the composite sample, including six Ag/Cu interfaces, increased by only 6.6%, indicating good conductivity of the Ag/Cu interface. Therefore, the obtained composite is a promising candidate for the fabrication of melt elements.

## 1. Introduction

Fuses have long been used in electrical installations to protect important equipment and human beings. Melt elements are the key components of fuses; they must melt and disconnect the faulty circuit portion once a sustained over-current or short-circuit current flows. For many applications, silver is the preferred material for melt elements due to its excellent electrical properties and chemical stability [1,2]. Recycling the expensive silver used in fuses is a challenge [3] because the volume of silver in the melt element is small, and fuses are typically dispersed among different systems and equipment. Copper is also a commonly used metal for melt elements in high-voltage applications [4,5], but the accuracy of fuses with copper can be lower due to its inferior oxidation resistance and electrical conductivity compared to silver. Therefore, it is a wise choice to decrease the consumption of Ag by developing a Ag/Cu through-layered composite, where Ag is used at the fused part to ensure accurate melting in case of an electrical accident, and copper is used in the rest of the composite for electrical and mechanical connections. In such composites, the Ag and Cu layers are bonded side-by-side, with the width of Ag matching the gauge of the fused part, so that the blanks of the fuses can be stamped from the composites.

Diverse techniques have been developed to join dissimilar metallic materials, such as continuous casting [6], rolling [7,8], explosion welding [9], stir friction welding [10], brazing [11], and diffusion welding [12,13]. Roll bonding is a well-accepted technique for the fabrication of Ag/Cu layered composites [14,15,16,17]. However, most of the layered composites are bonded on the wide faces with an up–down structure, including inlay and overlay composites, and they are not suitable for the fabrication of fuses. Recently, a Ag/Cu through-layered composite was fabricated by diffusion welding and hot-roll bonding [12,18,19], with the aim of developing a potential candidate for the melt elements.

The microstructure of the interface is a key concern due to its profound influence on the mechanical and physical properties of layered composites [20,21,22,23]. Fortunately, no brittle intermetallic compound, which usually damages the interface bonding, will be formed during the joining of Ag and Cu, according to the phase diagram. Instead, a solid solution with a different composition is formed in the diffusion layer across the interface of the Ag/Cu composite [14,15,24]. Secondary phase particles are found near the interface due to supersaturation at declining temperatures, but they are solid solutions also, i.e., Cu-enriched particles in the Ag layer and Ag-enriched particles in the Cu layer [25]. Both Ag and Cu have a face-centered cubic (FCC) lattice, and their lattice constants are 0.41 nm and 0.36 nm, respectively, differing by about 10%. Therefore, semi-coherent interfaces are usually formed between Ag and Cu phases on the same family of crystal planes, such as {111}Ag//{111}Cu and {100}Ag//{100}Cu, which are referred to as the cube-on-cube orientation [26,27], and the heterotwin structure on the {111}Cu//{111}Ag interface is another typical structure [28,29,30]. These interfacial characteristics are found in Ag/Cu alloys and composites that are prepared by casting and plastic deformation. However, it is relatively rare to study the interfacial structures of Ag/Cu layered composites fabricated under solid-state conditions. Ding [12] and You et al. [24] gave some evidence on the cube-on-cube orientation in Ag/Cu layered composites fabricated under solid-state conditions by diffusion welding and cross-accumulative roll bonding, respectively.

One of the key requirements for the application of layered composites is interface bonding strength. During roll bonding processes, the bonding mechanisms involve physical contact, metallic bonding, and metallurgical bonding, which are crucial for achieving high bond strength [31]. Therefore, surface properties, such as shape, hardness, and oxidation, influence the bonding strength significantly [32,33]. Interdiffusion between different layers is necessary for the formation of metallurgical bonds, but brittle intermetallic compounds are formed as a result in many cases. Therefore, interlayers are introduced to increase the bonding strength by controlling the diffusion mechanism [34] and improving the distribution and properties of intermetallic compounds [35,36]. Also, it is important to keep a low interface resistivity of the composites for electrical conductive applications [37]. As an electrical conductive composite, it is important to keep the interface resistivity of the Ag/Cu through-layered composite as low as possible. As reported by Liu et al. [18], hot-roll bonding increases the electrical resistivity of a Ag/Cu through-layered composite by 4% or so, while diffusion welding increases it by 6% or so. This can be attributed to the lattice distortion resulting from the interdiffusion of Ag and Cu atoms. When used as a melt element, an extra resistance heating effect will be introduced in the interdiffusion layer by repeated current impacting, and the melting temperature of Ag decreases with an increase in Cu content. Hence, the fusing feature and reliability will be influenced by the interdiffusion layer to some extent. Moreover, the design accuracy of melting elements can be affected by the width of the diffusion layers. Therefore, it is desirable to develop a Ag/Cu through-layered composite for melt elements with sufficient bonding strength (surpassing the tensile strength of Ag, which is lower than that of Cu), low interface resistivity, and a narrow interdiffusion layer.

In order to ensure the accurate fusing of the melt elements with the Ag/Cu through-layered composite, it is important to control the interdiffusion between Cu and Ag layers strictly, as long as a sufficient bonding strength is achieved. The width of the interdiffusion layer interface obtained by diffusion welding and one-step hot-roll bonding is 95–140 μm and 45–80 μm, respectively [18]. Thus, its application in melt elements is limited. In our previous work [38], we found that the interdiffusion layer is about 2 μm in width across the AgPd30/CuNi18Zn26 interface fabricated by the one-face roll-inlaying process. Based on this experience, a novel dual-face roll-inlaying technique has been developed for the first time to fabricate a Ag/Cu through-layered composite with limited interdiffusion layers, characterized by the bonding of Ag and Cu layers on the RD-ND section instead of the RD-TD section. The interfacial characteristics of the Ag/Cu composite have been investigated to evaluate the potential of this composite in the application of melt elements.

## 2. Brief Process of the Fabrication of Ag/Cu Through-Layered Composite

**Figure 1:**
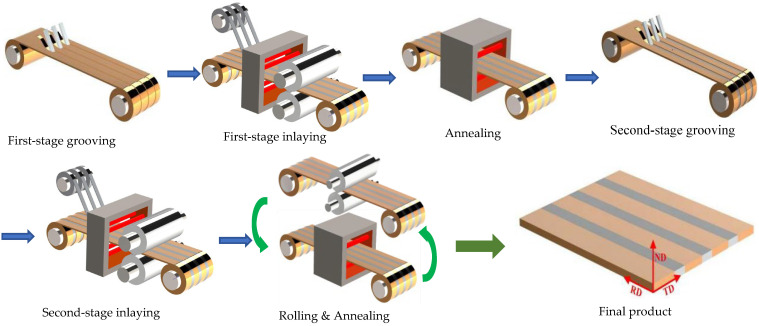
Schematic diagram of the production process of Ag/Cu through-layered composite.

Pure Ag (99.99 wt.%) and pure Cu (99.97 wt.%) strips are prepared by conventional rolling and annealing. In order to fabricate the Ag/Cu through-layered composite for use in the fabrication of melt elements for fuses, a novel dual-face hot-roll inlaying process has been developed, as shown in Figure 1. The main procedures are as follows:

1. A desired number of trapezoidal grooves are cut along the longitudinal direction on one surface of a Cu strip;
2. Ag strips are inlaid into the grooves by hot-rolling and named 1-Ag, leading to a layered composite with Ag inlaid in Cu;
3. The obtained Ag/Cu layered composite is annealed to improve the interface bonding strength;
4. Trapezoidal grooves similar to those cut in step 1 are grooved on the other surface of the as-annealed composites at the positions corresponding to the inlaid Ag layers to expose their bottom surface;
5. Additional Ag strips are inlaid into the grooves and named 2-Ag to obtain a through-layered Ag/Cu composite by hot-rolling;
6. The through-layered Ag/Cu composite is rolled and annealed to the specified dimension with enough interface bonding strength.

The blanks of melt elements will be stamped from the composite. Therefore, the composite should have appropriate strength and plasticity, which is realized by adjusting the final cold-rolling reduction to maintain a certain degree of work-hardening. That is to say, the final composite is supplied in the as-cold-rolled state. After each procedure, the composite strips are coiled, and continuous annealing [39]—instead of batch annealing—is applied. Therefore, this process is suitable for massive production of the through-layered Ag/Cu composite. In order to improve efficiency and decrease cost, a multilayered composite comprising up to ten layers of Ag can be produced.

The total reduction in Cu layer thickness is over 90%, that is, a reduction from the original thickness of 1.5 mm to a final thickness of 0.1 mm. Generally, the reduction and temperature values of hot-roll bonding are 30~40% and 400~500 °C, respectively, and the subsequent rolling and annealing are conducted with reduction values of 20~40% (except for the final cold-rolling) and temperatures of 500~600 °C, respectively. During continuous annealing, the composite is held at the highest temperature for 2~5 min. The bonding and rolling are conducted with a four-high rolling mill, and the Ag and Cu strips are protected by 75 at.% H_2_ + 25 at.%N_2_ during heat treatment. The detailed process parameters cannot be provided due to commercial confidentiality considerations.

## 3. Materials and Methods

In this study, only a composite with three Ag layers after final cold-rolling with 10% reduction was analyzed, because this is the raw material for the fabrication of a melt element.

### 3.1. Microstructure Characterization

The microstructure of the composites was observed using the TD-ND section. After being ground and polished with 0.5 μm diamond paste, the samples were polished further with a Gatan 697 argon ion polisher (GATAN, Berwyn, PA, USA). Then, the microstructure was investigated by electron backscattering diffraction (EBSD) using a field-emission scanning electron microscope (FE-SEM, JEOL JSM-7800F, JEOL, Tokyo, Japan), because it is hard to reveal the microstructure of Ag and Cu simultaneously by metallographic etching. The step size was 550 nm, and the indexing rate of the patterns was over 95%. An electron probe microanalyzer (EPMA, JXA-8530F Plus, JEOL, Tokyo, Japan) was used to reveal the composition distribution across the interface.

The crystal structure of the interface was characterized on a transmission electron microscope (TEM, FEI Talos F200S G2, FEI, Waltham, MA, USA). The accelerating voltage and aperture size were 200 kV and 220 nm, respectively. TEM samples were cut from the TD-ND plane with a DualBeam system (FIB, FEI Helios NanoLab 600i, FEI, Waltham, MA, USA), followed by ion thinning to about 50 nm for observation.

Additionally, the textures of the Ag and Cu layers were analyzed separately from the RD-TD plane (the surface) with an X-ray diffractometer (XRD, Rigaku D/max 2500PC, Rigaku, Tokyo, Japan). Cu Kα-radiation (λ = 1.5406 Å) was used, with a voltage of 40 kV and a current of 150 mA.

### 3.2. Mechanical and Electrical Properties Measurement

**Figure 2:**
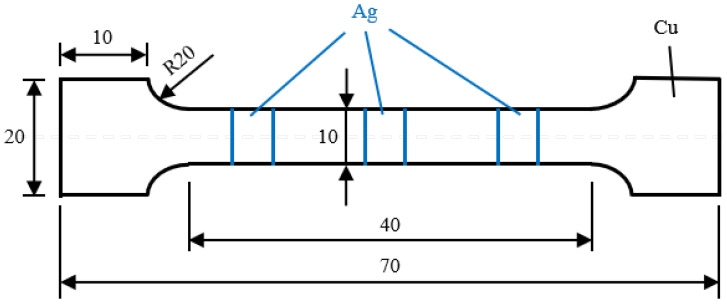
Schematic diagram of the tensile samples. All dimensions are in millimeters (mm).

As shown in Figure 2, three dog-bone tensile samples were machined from the final composite perpendicular to RD, so as to evaluate the interface bonding strength. According to Chinese National Standard GB/T 228.1-2010 [40], tensile tests were conducted on an A CMT-5105 material testing machine (SENS, Shengzhen, China), with a strain rate of 1.0 × 10^−3^ m/s. Continuous yielding was found in the samples; thus, the stress at 0.2% offset was taken to be the yield strength. Finally, the typical fracture surfaces were observed using a scanning electron microscope (SEM, Tescan Vega Ⅲ, Brno, Czech Republic).

Microhardness measurement was conducted on the surface with a HV-1000TM/LCD tester (HXT, Shanghai, China). The load and holding time were 0.98 N and 15 s, respectively. The 2-Ag/Cu interface of each Ag strip was measured because it was bonded at a later stage and, thus, was weaker than the 1-Ag/Cu interface. Also, a Ag and Cu matrix was measured for comparison. All the tests were repeated ten times, with five tests being performed on each interface of each Ag strip. The three measured interfaces were named Ag/Cu-Ⅰ, Ag/Cu-Ⅱ, and Ag/Cu-Ⅲ. Ag/Cu-Ⅱ was located on the middle Ag strip, while Ag/Cu-Ⅰ and Ag/Cu-Ⅲ were located on both sides.

For resistance measurement, ten rectangular samples were cut from the final composite perpendicular to RD, with effective dimensions of 14 (width) × 51 (length) mm. The resistance of the samples was measured using an AE-1152 DC low-resistance tester (AEMIC, Shizuoka, Japan) at room temperature via the four-point-probe direct current technique from the surface of the samples. The resistivity was calculated according the Ohm’s law. The theoretic resistivity of the composite was calculated with the following formula, using the Ag-Cu series circuit model and ignoring the effect of interdiffusion at the interface:

$$\rho ={(\rho }_{Ag}\times L_{Ag}+\rho_{Cu}\times L_{Cu})/L$$

where *ρ*, *ρ_Ag_* (1.64 × 10^−3^ mΩ·cm), and *ρ_Cu_* (1.72 × 10^−3^ mΩ·cm) are the theoretic resistivity and resistivity of Ag and Cu, respectively; *L*, *L_Ag_*, and *L_Cu_* are the length of the total sample and that of Ag and Cu strip, respectively (note that the length of these samples are the indeed the width of the composite).

## 4. Results

### 4.1. Ag/Cu Interfacial Microstructure

**Figure 3:**
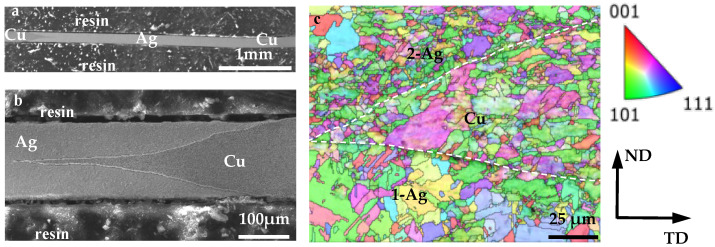
Morphology of (**a**) the cross-section of a Ag layer and (**b**) magnified interface region. (**c**) IPF image across the Ag/Cu interface (indicated by white dashed lines). 1-Ag and 2-Ag represent the Ag layers inlaid at the first and second stage, respectively.

Images of the overall cross-section of the Ag layer and the magnified morphology of the left interface region are shown in Figure 3a and Figure 3b, respectively. In order to improve the bonding strength, trapezoidal grooves were fabricated on the Cu strips, leading to V-shaped Ag/Cu interfaces in the through-layered composite. The microstructure of the interface region is shown in Figure 3c. No holes or cracks are found, indicating a well-bonded effect. The interface is flat, without obvious zigzags, unlike the zigzagged Ag/Cu interface fabricated by Liu et al. [18]. The grains in 1-Ag (inlaid at the first stage) are coarser than those in 2-Ag (inlaid at the second stage) but similar to those in the Cu layer. Moreover, fine grains are found at the interface, but there is no obvious fine-grained band in both the Ag and Cu layers.

**Figure 4:**
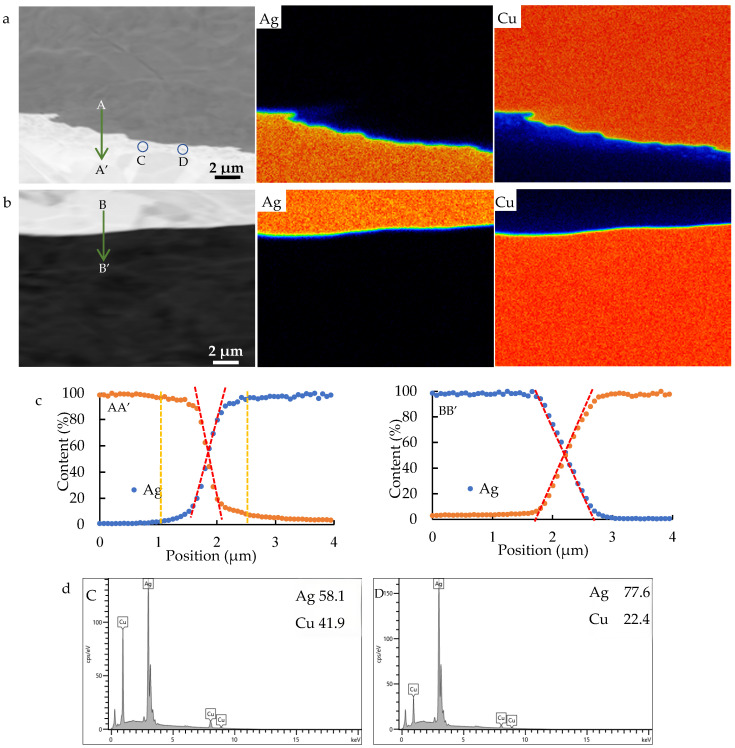
BSE image and EDS mapping of Ag and Cu across the interfaces of (**a**) 1-Ag/Cu and (**b**) 2-Ag/Cu. (**c**) Composition (at.%) distribution along arrow AA’ and BB’ and (**d**) composition (at.%) of particles C and D. The contents in the line-scanning profiles were calculated by comparing the counts of Ag and Cu measured at the interfaces with those from the matrix.

The compositional distribution of the interfaces is shown in Figure 4. The differences in interdiffusion performance across the two parts of the interfaces can be found in the EDS mapping (Figure 4a,b). At the 1-Ag/Cu interface, a Cu-containing band was formed in the Ag side, accompanied by a Ag-containing band in the Cu side, and the former is wider than the latter. At the 2-Ag/Cu interface, a Cu-containing band was formed in the Ag side, being much narrower and weaker than that at the 1-Ag/Cu interface. However, on the Cu side, a Ag-containing band was not observed. Moreover, some dots with Cu of greater intensity appear in 1-Ag near the interface, corresponding to the particles in the interface region of 1-Ag. Two particles were indicated by blue circles in the backscattered electron (BSE) image, which were determined by point composition analysis to be Cu-enriched phases (Figure 4d).

As shown by the red dashed lines overlapped on the line-scanning curves (Figure 4c), there are X-shaped zones resulting from the intersection of the composition distributions of Ag and Cu across the interfaces, where the contents of Ag and Cu change linearly. Dispersive diffusion zones (between the yellow and red dashed lines), where the contents of Ag and Cu decrease slowly with increasing distance from the interface, can be found in the 1-Ag/Cu interface but not in the 2-Ag/Cu interface. The X-shaped zone in the 1-Ag/Cu interface is narrower than that in the 2-Ag/Cu interface. However, the overall width of the interdiffusion region in the former is wider than that in the latter, provided the dispersive diffusion zones are taken into account. Generally, the widths of the Ag/Cu interdiffusion regions are less than 2 μm, much narrower than those in the Ag/Cu through-layered composite fabricated by hot-rolling (45–80 μm) and diffusion welding (95–140 μm) [18].

### 4.2. Crystallographic Orientation of Ag/Cu Composite

**Figure 5:**
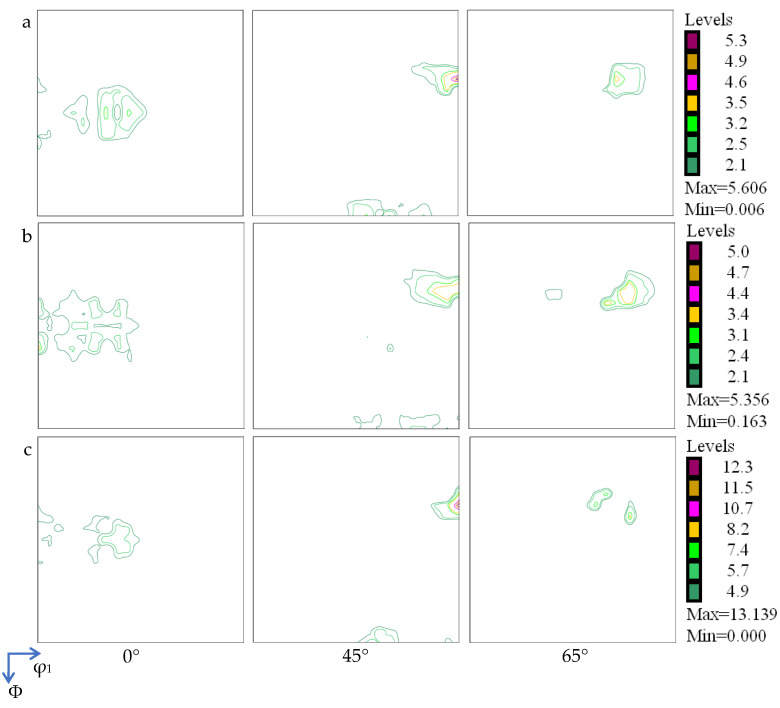
Orientation distribution function (ODF) plots (φ2 = 0, 45, 65°) of (**a**) 1-Ag, (**b**) 2-Ag, and (**c**) Cu.

The Ag/Cu through-layered composite was fabricated from three individual metal strips, which are 1-Ag, 2-Ag, and Cu. Thus, their textures were analyzed separately. Employing Bunge’s notation for the Euler angles, Figure 5 shows sections of these samples with the following properties: φ2 = 0°, φ2 = 45°, and φ2 = 65° ODF. The same textural components were found in the three layers, including copper ({112}<111>), brass ({110}<112>), and S-type ({123}<634>) components [41,42,43]. The textural intensities of the 1-Ag and 2-Ag layers are close to each other (around 5.5), although they were inlaid into the composite at different stages. Overall, the texture in the Cu layer is much stronger (above 13.0).

**Figure 6:**
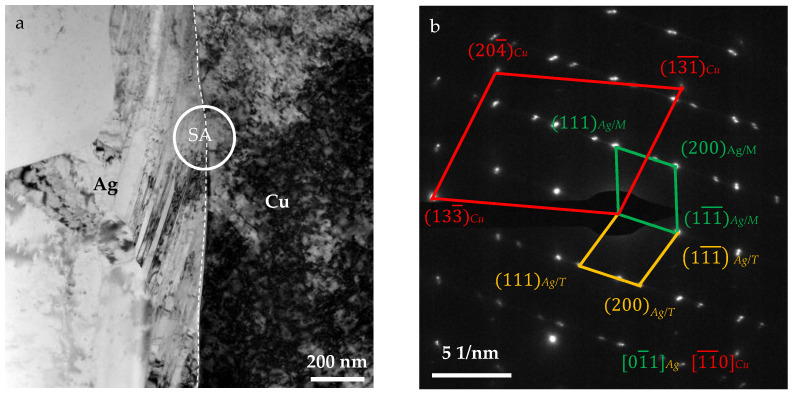
(**a**) TEM image and (**b**) SAED pattern of SA (the white circle) at the 1-Ag/Cu interface. The white dashed line shows the 1-Ag/Cu interface.

Due to its longer rolling duration and annealing time, the 1-Ag/Cu interface is believed to develop a better orientation relationship than the 2-Ag/Cu interface. This was observed under a TEM, as shown in Figure 6. The interface is clear and smooth (Figure 6a), but no well-matched crystallographic orientation relationship was detected by selective area electron diffraction (SAED; Figure 6b). In addition, many twins were found in the Ag side (Figure 6a), and a {111}<110> twin was identified by SAED (Figure 6b).

**Figure 7:**
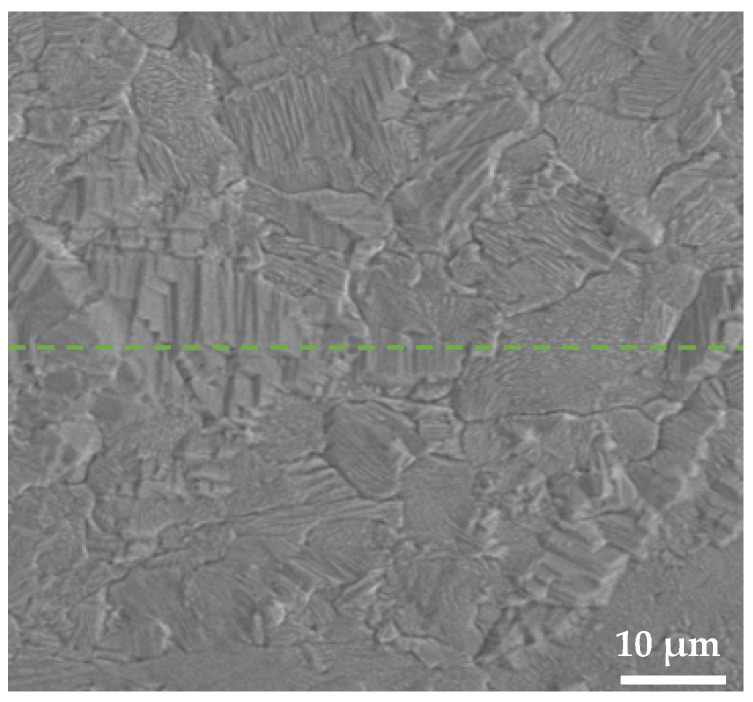
SEM microstructure of the Ag layer across the disappeared interface between 1-Ag and 2-Ag. The dashed line implies the speculative position of the original Ag/Ag interface according to the top of the V-shape shown in Figure 3.

During the present compositing process, the Ag-layer was composed of two separate strips at the beginning, i.e., 1-Ag and 2-Ag. Surely, a Ag/Ag interface was formed after the inlaying of 2-Ag, which can be determined by connecting the tips of the V-shaped Ag/Cu interface (Figure 3a). Interestingly, no interface can be found between them in the final composite (Figure 7), indicating that the grains grew across the Ag/Ag interface and merged together as a result of mechanical bonding, self-diffusion, and recrystallization occurring during the fabrication process.

### 4.3. Mechanical Properties and Resistance

**Figure 8:**
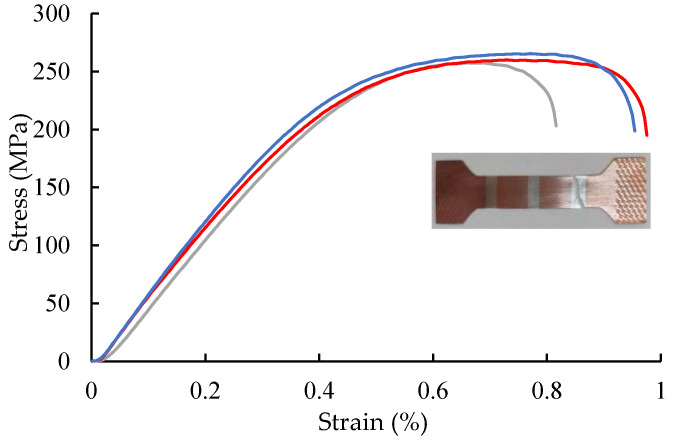
Tensile stress–strain curves and a typical image of the samples.

Figure 8 presents the engineering stress–strain curves of the three Ag/Cu composite samples, along with a typical image of the broken samples. The average yield strength, tensile strength, and elongation are 113 ± 2 MPa, 260 ± 5 MPa, and 0.92% ± 0.08%, respectively. Obvious drops appear at the end of the stress–strain curves, suggesting the shrinkage of the fracture surface. All three tensile samples fractured in the Ag layer rather than at the Ag/Cu interface (inset in Figure 8), implying that the Ag/Cu interfacial bonding strength surpasses the tensile strength of the Ag layer.

**Figure 9:**
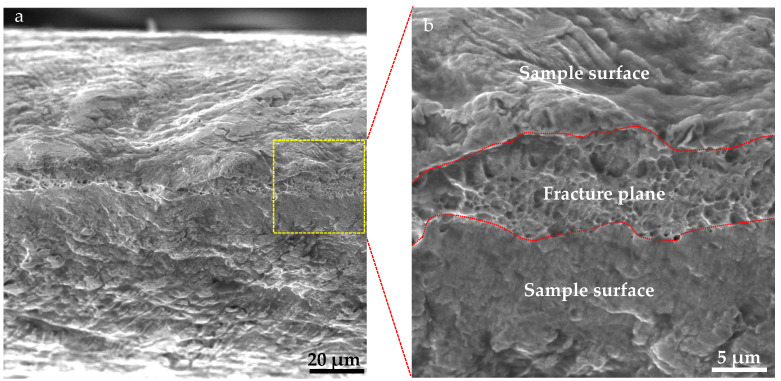
Tensile fracture surface morphology at (**a**) low and (**b**) high magnifications. The dashed lines indicate the fracture plane.

Figure 9 shows the tensile fracture morphology of the Ag/Cu composite. As shown in Figure 9a, the width of the fracture surface is generally less than 10 μm, decreasing by over 90% compared with the original thickness. Plastic deformation features, such as folds and waved traces, can be seen on the sample surface (the RD-TD plane), being adjacent to the fracture surface. At higher magnification (Figure 9b), dimples are observed all over the fracture surface, indicating the excellent ductility of silver.

Table 1 shows the microhardness of the samples. Not surprisingly, the microhardness of all the Ag/Cu interfaces is higher than that of Ag but lower than that of Cu, indicating that the Ag/Cu bonding strength falls between the strength of the Ag and Cu matrices. Therefore, the tensile samples were broken in the Ag layer.

The average resistance of 10 measurements taken from the Ag/Cu through-layered composite is listed in Table 2, along with the calculated results. By comparing the measured resistivity with the calculated values, it can be found that the existence of Ag/Cu interfaces causes an increase in resistivity of about 6.6%. There are 6 V-shaped Ag/Cu interfaces in each sample; thus, the resistivity was increased by 1.1% due to the formation of one Ag/Cu interface.

## 5. Discussion

### 5.1. Interdiffusion Between Ag and Cu Layer

Ag and Cu form a simple eutectic alloy, without any intermetallic compound after solidification. Instead, Ag-enriched and Cu-enriched solid solutions are formed. As the temperature decreases, the solubility of Cu in Ag-enriched solid solution decreases to almost 0 at 200 °C, and vice versa. Hence, a Ag/Cu composite can be fabricated in situ using melting techniques, followed by severe plastic deformation methods, including cold-rolling, accumulative roll-bonding, and high-pressure torsion [28,29,44]. For such composites, the metallurgical Ag/Cu interface bonding is formed due to the eutectic decomposition upon solidification. Additionally, the solid-state diffusion across the interface decreases the concentrations of the two solid solutions. However, the components, Ag and Cu, are distributed in these composites dispersively, although a layered structure can be obtained. Obviously, this technique is not suitable for manufacturing layered composites for use in the fabrication of melt elements.

Solid/liquid hybrid [22,45,46,47] and solid-state bonding [8,48,49] are commonly used to produce macro-scale layered composites. During solid–liquid composite processes such as continuous casting, a metallurgically bonded interface can be achieved by melting bonding and diffusion bonding during the first bonding procedure, because of the high temperature and the sufficient wetting of the solid component by the liquid [50]. Also, solid-state bonding processes based on diffusion welding [12] can achieve a metallurgically bonded interface at the first stage because sufficient diffusion occurs in the interface region to form an alloy layer. Based on these processes, working plastic is applied to adjust the geometric dimensions primarily. However, in hybrid processes, a Ag-Cu eutectic microstructure can be formed at the interface [45], thus rendering the composite not suitable for use in melt elements because of the interface’s very low melting temperature. On the other hand, the width of the Ag/Cu interdiffusion layer in the composite obtained from diffusion welding is over 100 μm [18], limiting the application of the Ag/Cu composite for use in the melt element fabrication for fuses.

During solid joining processes such as hot-rolling, only partially metallurgical bonding can be achieved in the initial interface, which will be improved gradually by the elemental interdiffusion across the interfaces in the subsequent procedures, including rolling and annealing [38,51]. Therefore, the width of the interdiffusion layer can be controlled effectively while the interface is well bonded. In fact, the width of the interdiffusion layer of the Ag/Cu through-layered composite fabricated by one-step hot-roll bonding is 45–80 μm, about half of its counterpart fabricated by diffusion welding [18].

**Figure 10:**
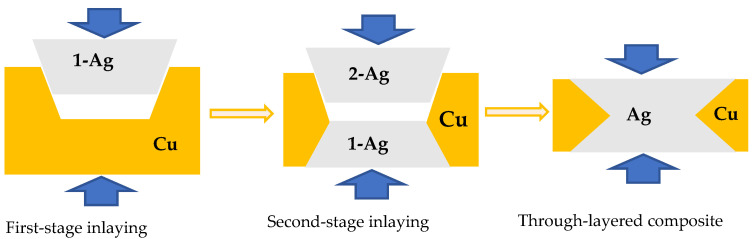
Schematics of the two-step inlaying technique developed in this work. The blue arrows indicate the rolling pressure.

In this work, a two-step inlaying technique was developed. Ag strips with a trapezoidal cross-section were fit into the trapezoidal grooves in the Cu strip during both steps, as shown in Figure 10. During the bonding procedure, the sides were subjected to a portion of the rolling pressure, along with the lateral pressure, and thus, the sides of Ag strips were bonded with Cu effectively at the initial stages. During the subsequent rolling, the Ag/Cu interfaces were further subjected to the rolling pressure, due to the development of the matched trapezoidal sides. Therefore, the rolling process contributed significantly to the Ag/Cu bonding strength. Furthermore, the Ag/Cu interdiffusion was controlled carefully by adjusting temperature and holding time (moving speed of the composite strip) during the continuous annealing process. As a result, the width of the interdiffusion layer was controlled under 2 μm (Figure 4), while the bonding strength surpassed the tensile strength of Ag (Figure 8).

The Ag layers in the final composite are composed of two sub-layers, 1-Ag and 2-Ag. Compared to the 1-Ag/Cu interface, the 2-Ag/Cu interface was formed later, and thus, it underwent shorter interdiffusion periods. Differences in the interdiffusion regions can be found in Figure 4. Generally, the 1-Ag/Cu interdiffusion region is wider than the 2-Ag/Cu one. X-shaped zones are found in both interfaces, while the dispersive diffusion zones appear in the 1-Ag/Cu interface only. Elemental interdiffusion between the Ag and Cu layers consists of two steps. Firstly, Ag and Cu atoms jump across the interface into the other layer, forming the X-shaped zones in the EDS line-scanning profiles. Secondly, these atoms migrate from the interface to the matrix, leading to the dispersive diffusion zones. Obviously, the second step is slower than the first step, and therefore, it can be deduced that the development of the Ag/Cu metallurgical bonding during solid-state joining is a diffusion-controlled process instead of an interface-controlled one. From this point of view, the initial mechanical bonding is vital for the bonding strength of the Ag/Cu through-layered composite with a strictly controlled interdiffusion layer.

Observing the composition distribution profiles carefully, it can be noted that the diffusion distance of Cu in Ag is longer than that of Ag in Cu, especially in the 1-Ag/Cu interface. This is because of the faster migration rate of Cu in Ag [17,45]. The solubility of Cu in Ag decreases with decreasing temperature. Therefore, Cu-enriched particles were deposited from the Ag-based solution during the cooling process after annealing and hot-rolling. Fortunately, the decomposition of the solid solution is conducive to lowering the electric resistivity and inducing precipitation strengthening. Theoretically, Ag-enriched particles should be formed in the Cu layer near the interface, but they were not found in this work because they are smaller and fewer in number compared to the Cu-enriched particles.

Diffusion occurred between 1-Ag and 2-Ag. This was indeed a self-diffusion process, and thus, it did not induce the change in composition. At the same time, grains at the interface grew into the other side, and some of them coalesced with each other. As a result, the interface disappeared after multiple passes of rolling and annealing.

### 5.2. Effect of Microstructure on Bonding Strength and Electrical Resistivity

During the bonding processes, the composite underwent repeated deformation and recrystallization, and the microstructure evolved accordingly. At the interface, additional shear stress occurred during the rolling procedure due to the difference in plasticity between Cu and Ag, leading to finer grains in both layers. Moreover, the grain growth in the interdiffusion region can be restrained by solute atoms and secondary-phase particles resulting from diffusion and precipitation, and thus, fine-grain bands were found at the Ag/Cu interface by Ding [12] and Meng et al. [16]. In this work, finer grains were found at the interface without “band” features, attributed to the limited interdiffusion. Usually, grain boundaries increase the electrical resistance [52], but the bonding strength can be improved by strengthening fine grains. In addition, before the secondary step, inlaying of 2-Ag, the inlaid 1-Ag/Cu was annealed to increase the bonding strength. Therefore, the 1-Ag and Cu layers underwent one more annealing process than the 2-Ag layer, leading to the coarser grain size in the former.

Both Cu and Ag are FCC metals, and thus, they usually have similar textures after plastic deformation, including copper, brass, and S-type components [41,42,43], as identified in this work. In the work of Liu et al. [37], copper- and brass-type textures were found in both the Ag and Cu layers of a bi-layered Cu/Ag composite after rolling to thickness reductions of 80% and above. Additionally, the textural composition is affected by stacking fault energy (SFE). In FCC metals with medium to high SFE (>30 mJ/m^2^), the copper and S texture evolves dominantly. However, those with low SFE (<30 mJ/m^2^) show a stronger tendency to form a brass-type texture [43,49]. The SFE of Cu (45 mJ/m^2^) is higher than that of Ag (26 mJ/m^2^). Hence, the intensity of the copper-type texture in the Cu layer (about 13.0) is much stronger than that in the Ag layer (about 5.5). Moreover, the stronger texture in the Cu layer can be ascribed to its higher crystallization temperature.

Due to the shared lattice and similar lattice constant, the cube-on-cube orientation is usually found between Ag and Cu phases in Ag-Cu alloys and composites [26,27,28,29,30]. Considering the same texture compositions in Ag and Cu layers, the cube-on-cube orientation, including Ag(100)/Cu(100), Ag(110)/Cu(110), and Ag(111)/Cu(111), should probably be developed in the present Ag/Cu through-layered composite. In fact, Ding et al. [12] determined these orientation relationships in a Ag/Cu bimetallic strip fabricated by diffusion welding based on the texture analysis of the different layers. Furthermore, in the multinanolayered Ag/Cu composite prepared by cross-accumulative roll bonding, the Cu and Ag grains are found to be oriented with their respective {110} and {111} planes parallel to each other, resulting from the extreme rolling deformation [24]. In this work, the through-layered composite was fabricated in the solid state with a very thin diffusion layer (less than 2 μm). The orientations of the grains beside the interface, found in the lateral surfaces of the Ag and Cu strips before inlaying, were unrelated to each other initially. Inevitably, lots of grains with random orientation relationships were bonded together at the interface. In the subsequent procedures, the orientations of grains can only be adjusted by deformation and recrystallization, but they may be insufficient. Moreover, the recrystallization and grain growth were hindered by the solute atoms and second-phase particles resulting from the interdiffusion. Therefore, the crystal orientation of Ag and Cu grains next to the interface was not adjusted sufficiently to achieve a well-matched microstructure. As a result, the cube-on-cube Ag/Cu orientation relationship was not detected by SAED investigation. At the macro scale, the crystallographic orientations of Ag and Cu layers are generally the same, while in the micro-scale, well-matched orientation, relationships are not established sufficiently at the interface.

Grains at the interface are finer than those in the matrix, leading to fine-grain strengthening. The elemental interdiffusion at the interface resulted in solution- and precipitation-strengthening effects. Moreover, the V-shaped interface increases the bonding area and load-bearing ability. Therefore, the interface bonding strength is higher than the ultimate strength of Ag, 260 MPa, as revealed by tensile testing. On the other hand, the excellent Ag/Cu interface bonding strength was also confirmed by microhardness measurement. Interestingly, the section shrinkage is over 90% (Figure 9), while the elongation is less than 1%. It is well known that, under the same annealing condition, the strength of pure Cu is higher than that of pure Ag. And under the same reduction, the recrystallization temperature of Cu is higher too. In this composite, Cu layers have higher strength, and thus the plastic deformation is concentrated in Ag layers. The section shrinkage reflected the deformation of the fractured Ag layer, but the elongation was calculated based on the overall length of the sample. Thus, a large gap between these two properties appears. It is clear that the plasticity of the Ag layers is much better than that illustrated by the tensile curves, as confirmed by the dimples shown on the fracture plane.

As shown in Table 2, interfacial electrical resistivity is found due to the compositing process, and this can be attributed to several factors. Obviously, scattering of the Ag/Cu interface is the primary cause of the increase in electrical resistivity [23]. Solute atoms and secondary-phase particles, resulting from the elemental interdiffusion, contribute to the increasing resistivity attributable to the formation of lattice deformation and phase boundaries. Moreover, the finer grains next to the interface increase the resistivity to some extent by the formation of more grain boundaries as compared with the matrix [52]. In this work, the width of the interdiffusion layer is less than 2 μm, and therefore, it is hard to decrease the interface resistivity by controlling the interdiffusion further. In order to reduce the interface resistivity, it is advisable to optimize the interface structure via the following methods:

(1) Decreasing the length of the edges of the V-shaped interface under the condition of obtaining sufficient bonding strength;
(2) Developing a well-matched cube-on-cube crystal orientation relationship and a semi-coherent Ag/Cu phase boundary at the interface, which is also beneficial for increasing bonding strength [53].

## 6. Conclusions

The proposed dual-face hot-roll inlaying technique is effective for fabricating Ag/Cu through-layered composite. V-shaped interfaces are formed between Ag and Cu layers because the Ag strips with trapezoidal cross-sections were fit into the trapezoidal grooves in the Cu strip from the opposite surfaces. The two sub-layers of each Ag layer were integrated together completely without an “interface”.

Limited elemental interdiffusion occurred at the Ag/Cu interface, leading to a diffusion layer with a width less than 2 μm. The composition distribution across the interfaces formed at different stages showed different characteristics. Sharp variations in Ag and Cu concentrations were detected through EPMA analysis across the 1-Ag/Cu and 2-Ag/Cu interfaces. However, dispersive diffusion zones and Cu-enriched precipitations were found at the 1-Ag/Cu interface only.

The same textural components—copper, brass, and S-type components—have been developed in the Ag and Cu layers, but no well-matched crystallographic orientation relationship was identified via SAED between the Ag and Cu grains at the interface. The texture intensity of the two Ag sub-layers is similar, while that of the Cu layer is much higher.

The Ag/Cu interface bonding strength surpasses the tensile strength of Ag, i.e., 260 MPa, while each Ag/Cu interface contributes an increase of 1.1% to the electrical resistivity of the composite. Therefore, this Ag/Cu through-layered composite can be considered a suitable candidate for use in the fabrication of melt elements in fuse production.

## Figures and Tables

**Table 1:** Microhardness of the interface and matrix.

Position	Ag/Cu-Ⅰ	Ag/Cu-Ⅱ	Ag/Cu-Ⅲ	Cu	Ag
HV	71.2 ± 4.3	73.2 ± 5.5	72.3 ± 4.8	60.9 ± 3.5	111.4 ± 6.6

**Table 2:** Comparison of measured and calculated resistivity.

Total Width of Different Layers/cm		Measured Resistance /mΩ	Measured Resistivity /mΩ·cm	Calculated Resistivity /mΩ·cm	Deviation /%
Ag	Cu				
1.02	4.08	0.63	1.78 × 10^−3^	1.67 × 10^−3^	6.59

## Data Availability

The original contributions presented in this study are included in the article. Further inquiries can be directed to the corresponding author.
